# Factors influencing parent-child relationships in chinese nurses: a cross-sectional study

**DOI:** 10.1186/s12912-023-01413-7

**Published:** 2023-08-09

**Authors:** Lei Huang, Xia Huang, Jingjun Wang, Fengjian Zhang, Yang Fei, Jie Tang, Ya Wang

**Affiliations:** 1 Department of Nursing, West China Hospital, Sichuan University / West China School of Nursing, Sichuan University, Chengdu, 610041 Sichuan P.R. China; 2 School of Nursing, Tongji Medical College, Huazhong University of Science and Technology, Wuhan, 430030 Hubei P.R. China; 3 Department of Nursing, Dazhou Central Hospital, Dazhou, 635000 Sichuan P.R. China; 4 Mental health center, West China Hospital, Sichuan University / West China School of Nursing, Sichuan University, Cheng Du, Si Chuan P.R. China

**Keywords:** Nurse, Nurse-patient relationship, Parent-child relationship, Cross-sectional study, Factor

## Abstract

**Background:**

With the development of the social economy, the effective coordination of the conflict between work and family has become an urgent problem for most parents. Such conflicts are especially acute in the families of nurses with children. Therefore, a timely understanding of the status quo of the parent-child relationship and associated risk factors among nurses will assist in improving their family harmony and the healthy growth of their children.

**Methods:**

A total of 350 nurses with children at a general tertiary hospital in Sichuan Province, China, were interviewed using a structured questionnaire between June 23 and July 9, 2022. The results were analyzed by multiple linear regression using the stepwise method.

**Results:**

The results showed that the parent-child relationship received a middle-level mean score of 77.74 (SD = 10.77). The factors that influenced the parent-child relationship among nurses included the parents’ character type (β = 0.143, P = 0.002), feeling tired due to dealing with patients (β=-0.150, P = 0.002), the nurse-patient relationship (β = 0.137, P = 0.004), the age of older children (β=-0.153, P = 0.001), number of children (β=-0.093, P = 0.041), sleep quality (β = 0.116, P = 0.014), and family adaptability (β = 0.308, P = 0.000); these factors accounted for 31.3% of the variance in parent-child relationships among nurses.

**Conclusion:**

The findings of this study will help policy makers and nursing managers to better understand parent-child relationships in Chinese nurses. The results highlighted the importance of the creation of a family-oriented work environment while paying more attention to the parent-child relationships of nurses who are introverted and have more or older children. After busy workdays, nurses should also be encouraged to participate more in family decision-making and strategic parent-child interactions to avoid negative effects on children caused by work-related emotional exhaustion, physical and mental fatigue, and other reasons. The development of good parent-child relationships may help maintain both their and their children’s mental health while enhancing their enthusiasm for work and their professional identity.

## Background

With the development of social economy and culture, the connections between families and the labor force have changed dramatically [1]. The increased participation of couples in the labor force has been one of the most significant changes in the workplace in recent decades [2]. Thus, parents find it more difficult to effectively manage tasks and demands in both the work and family domains [1]. Nowadays, most parents face the dilemma of allocating time and energy between work and family responsibilities to coordinate work and family responsibilities. Such conflicts are especially acute in families with children [3]. Moreover, the extension of working days, commuting times, and increases in work pressure have placed considerable stress on the family compared with those experienced in the past [4].

Work-family conflict is a common and visible phenomenon, particularly in the nursing profession [5]. In contrast to other dual-income families, nurses tend to have a more complex work environment. Even with high-intensity workloads and closed and oppressive work environments, nurses must ensure that patients receive the highest quality nursing services [6, 7], as well as having to deal effectively with interpersonal relationships in the work environments [8]. The increased stress and energy exhaustion will inevitably affect the balance between the work and family lives of nurses. This is aggravated by the often irregular schedules and night shifts which deprive nurses of the opportunity to spend time with their families [9, 10]. A previous study has shown that non-standard work schedules of mothers adversely affect the growth of children [11]. In addition to clinical work, some medical institutions also require the completion of additional scientific research and teaching tasks [12]. This will inevitably occupy their leisure time, thereby reducing the time available to spend with their families, as well as their energy and enthusiasm for family interactions. Available studies have shown that between 81% and 93% of nurses experience challenges in handling family problems due to work-family conflicts [13, 14]. Under these circumstances, it is difficult for them to perform well at family functions, impacting nurses’ marital satisfaction and parent-child relationships [15, 16].

Parents who devote more time and energy to their jobs neglect their children’s care and education [4]. According to several reports, longer work hours are associated with reduced parent-child time, while high job requirements are related to lower levels of parent-child interaction and higher levels of conflict [3, 17]. Notably, the negative effects on children may be irreversible. Since ancient times, families have served as a haven for people’s growth [18]. A harmonious family environment is essential for a child’s healthy growth and good social adaptation [19]. In this case, parents should pay more attention to parent-child relationships, minimizing the interference of the external environment, interacting with their children, and establishing reliable contacts [20]. Previous research has shown that a close and harmonious parent-child relationship improves communication between parents and children, increases parenting efficiency, and provides children with opportunities to learn from their parents’ behavior and practice social skills [16]. Conversely, conflicts and poor parent-child relationships can aggravate children’s behavioral problems and jeopardize their mental health [21, 22], which is not conducive to children’s growth and personality development, particularly during the critical preschool years [23]. Meanwhile, a poor parent-child relationship may also impact parents’ work enthusiasm, professional identity, and health [24, 25].

To date, although there have been numerous reports on the influences on parent-child relationships, there are few studies on the experience of parents in managing their work and family roles and how this affects parent-child relationships [3]. Several studies have explored the impact of parental behavior on children from the perspective of adults. For example, Vieira et al. (2016) reported that the parent-child relationship determines how parents balance work and family, which has a different relationship with the children’s behavior [26]. Day et al. (2021) showed that certain behavioral patterns of parents, such as increasing the use of forced parenting, can lead to poor parent-child relationships [27]. A study conducted on 231 mothers with special-needs children in Australia found that increased stress, anxiety, and depression in mothers were predictive of poor parent-child relationships, and positive parenting experiences were the largest predictor of good parent-child relationships [28]. In addition, several other studies have shown that personal factors (e.g., age, education, income), environmental conditions (e.g., place of residence, employment status), family functioning (e.g., marital satisfaction, parental complementarity), and other factors can all affect individual parent-child relationships [23, 29, 30]. However, on the one hand, research on parent-child relationships at home and abroad has chiefly concerned the public [31] and has not addressed the impact of specific negative work experiences such as relationships with customers and work intensity on parent-child relationships. On the other hand, associations between certain personal traits (such as personality, lifestyle), family relationships (such as relationships with spouse’s parents), and other factors and parent-child relationships have not been examined. Furthermore, in terms of the family atmosphere, our previous research showed that although nurses bear high physical and emotional loads, their family cohesion and adaptability are generally better than the national average [32]. Good family cohesion helps to establish emotional connections among family members, while good family adaptability reflects the family’s ability to respond flexibly to new obstacles or stressful events by alteration of power structures and relationship rules [33]. Therefore, we hope that additional in-depth research can confirm the positive impact of family cohesion and adaptability, which are important manifestations of family functions, on the parent-child relationships of nurses.

In summary, this study aimed to explore the status and influencing factors among Chinese nurses. The important findings of this study will provide a scientific foundation for nursing managers to develop family-oriented work policies, especially on how to improve relationships with children, ensuring the physical and mental health development of the children of employees and helping nursing workers avoid the harm to work and family caused by children’s problems.

## Methods

### Design

A cross-sectional survey was used to investigate factors affecting parent-child relationships, as well as related factors contributing to the development of the parent-child relationship and their application to nurses.

### Samples

A convenience sample of nurses participated in a cross-sectional survey. Inclusion criteria: (1) registered nurses who were informed of the purpose of this study and voluntarily participated in this study; (2) nurses with children. Exclusion criteria: (1) intern or refresher nurses; (2) nurses with a child under the age of two, based on the applicability of the scale and Piaget’s theory on cognitive development [34, 39]. To ensure an appropriate sample size for the linear multiple regression analyses [35], G*power version 3.1.9.7 was used; assuming a significance level of 0.05, a median effect size of 0.15 [35], a power of 95%, and 20 predictors, the sample size was calculated to be 222 participants. Allowing for a sample loss rate of 20%, at least 278 participants needed to be surveyed. A total of 350 questionnaires were eventually completed, with a survey response rate of 97.2%. It was estimated that participants would take 10–15 min to complete the questionnaire.

### Measures

The general characteristics of participants included their gender, age, marital status, education, character, economic situation, relationship with the parents of the spouse, number of own children, the age of younger children, the age of older children, sleep quality, exercise frequency, and work-related information, including work seniority, post, the form of employment, department, nurse-patient relationship, whether they felt overworked while dealing with nurse-patient relationships.

The Family Cohesion and Adaptability Scale was used to measure family functioning in nurses [36], translated into Chinese by Fei [37]. This scale reflects the emotional ties that exist within families as well as the capacity of the family system to adapt to changing circumstances and issues at various stages of family development [32]. The scale has a total of 30 items, including two subscales of cohesion and adaptability. Each item is measured using a five-point Likert scale ranging from 1 (never) to 5 (very often). Good internal consistency has been reported, with a Cronbach’s α of family cohesion = 0.85 and a Cronbach’s α of family adaptability = 0.73 [36]. Family cohesion scores range from 28 to 92, while family adaptability scores range from 14 to 70. In this study, Cronbach’s α of family cohesion was 0.91, while for family adaptability, α = 0.79.

The Parent-Child Relationship Scale was used to measure the parent-child relationships between nurses and their children [38], developed by Ting [39]. The scale has been widely used in China and has undergone many revisions. The scale consists of two versions: the adult version and the children’s version, and this study only used the adult version to investigate the subjective feelings of parents about parent-child relationships between children and parents. The scale has 20 items in total. Each item is measured using a five-point Likert scale that ranges from 1 (very inconsistent) to 5 (very consistent). Good internal consistency has been reported, with a Cronbach’s α = 0.90 [39]. Possible scores range from 20 to 100, with higher scores indicating a better parent-child relationship. A total score of less than 60 on the scale suggests a severe problem in the parent-child relationship that calls for immediate adjustment. Scores between 60 and 80 points indicate that, although the parent-child relationship is good at present, it nevertheless requires adjustment, while a total score of more than 80 points suggests that the parent-child relationship is good and can be maintained. Cronbach’s α in this study was 0.92.

### Data collection

Data for this study were collected from a general tertiary hospital in Sichuan Province, China, between June 23 and July 9, 2022. The purpose and significance of this study were thoroughly explained to the nurses in the departments by the investigators after obtaining approval from the nurse managers of the selected departments. Subsequently, the electronic questionnaires were sent to the nurse managers of the departments, who then distributed them to the nurses within the departments who met the inclusion and exclusion criteria. The investigation was conducted following the principles of voluntariness and anonymity and all nurses participating in the study were required to sign an informed consent form. The online questionnaire was completed using mobile devices running the WeChat app. After completing the survey, the personnel of the survey team checked whether the data were missing or filled out in disorder and eliminated the questionnaires that did not meet the requirements. The data were then entered into the database by the research team.

### Data analysis

The collected data were analyzed using SPSS version 19.0 (IBM Corp., Armonk, NY, USA). The composition ratio (IQR) and mean (SD) were used to describe demographic data, parent-child relationships, and other scale scores. Independent-sample t-tests, one-way analyses of variance, and Pearson’s correlation analysis were used to compare the differences between different groups of influencing factors. Multiple linear regression analysis using the stepwise method was conducted to investigate the factors influencing participants’ parent-child relationships. The residuals from the models were tested using the K-S test and evaluated in combination with the histograms and Q-Q plots, indicating a normal distribution. Variance inflation factor values were checked, with all values being below 1.2, indicating no multicollinearity problems. A subsequent residual analysis showed a Durbin-Watson value of 1.94, verifying independence of the residuals. The overall significance levels were set at P < 0.05.

### Ethical considerations

The study was approved by the Ethics Committee on Biomedical Research, West China Hospital, Sichuan University (Ethical review No. 2020 − 333). Participants’ identity information was anonymized to protect their personal information. Informed consent was obtained from all subjects.

## Results

### Status and univariate analysis of parent-child relationship in nurses

The median age of the study participants was 34 (IQR: 30–38), and 336 (96.0%) were female. Most participants were married (88.3%) and had a bachelor’s degree (73.4%). The younger children of the participants had a median age of 4 (IQR: 2–7), while the older children had a median age of 6 (IQR: 3–10). The mean score for the parent-child relationship was 77.74 (SD = 10.77). Univariate analysis revealed significant differences in a parent-child relationship in terms of character, relationship with the parents of the spouse, number of own children, sleep quality, exercise frequency, nurse-patient relationship, feelings of tiredness after dealing with patients, and feelings of being overworked (P < 0.05), as shown in Table 1. The results of correlation analysis showed strong negative correlations between the age of older children and the parent-child relationship (r=-0.171, P = 0.001), family cohesion and the parent-child relationship (r = 0.416, P < 0.001), and family adaptability and the parent-child relationship (r = 0.447, P < 0.001). Meanwhile, age and the parent-child relationship showed a weak negative correlation (r=-0.116, P = 0.030). However, no correlations were found between the age of younger children and the parent-child relationship (r=-0.090, P = 0.093) or between work seniority and the parent-child relationship (r= -0.073, P = 0.174), as shown in Table 2.

**Table 1:** Comparison of demographic data of parent-child relationships (N = 350)

Variables	N	Mean (SD)	F/t	P
**Gender**			-0.769^a^	0.442
Male	14	75.57 ± 8.55		
Female	336	77.83 ± 10.86		
**Marital status**			0.928^b^	0.396
Unmarried	24	78.87 ± 12.05		
Married	309	77.83 ± 10.60		
Divorced or widowed	17	74.47 ± 12.09		
**Education**			1.600^b^	0.203
College degree or under	75	75.84 ± 10.34		
Bachelor	257	78.18 ± 10.95		
Postgraduate or above	18	79.39 ± 9.44		
**Character**			10.653^b^	0.000*
Introverted	102	75.60 ± 11.30		
Extroverted	164	80.49 ± 9.90		
Uncertain	84	74.99 ± 10.56		
**Economic situation**			1.816^b^	0.164
Poor	56	79.07 ± 10.59		
Common	179	78.30 ± 11.02		
Good	115	76.23 ± 10.38		
**Relationship with spouse’s parents**			9.248^b^	0.000*
Inharmonious	10	67.50 ± 10.69		
Common	120	75.76 ± 10.96		
Harmonious	220	79.29 ± 10.28		
**Number of own children**			3.164^b^	0.043*
1	230	78.53 ± 10.66		
2	92	75.35 ± 10.70		
Others	28	79.14 ± 11.10		
**Post**			0.802	0.449
Responsible nurse	221	77.62 ± 10.95		
Nursing team leader	79	78.89 ± 10.68		
Head nurse or above	50	76.48 ± 10.12		
**Form of employment**			0.854^a^	0.394
Contract labour	289	77.97 ± 11.08		
Permanent staff	61	76.67 ± 9.17		
**Department**			1.171^b^	0.323
Medicine department	50	77.92 ± 10.23		
Surgical department	72	78.49 ± 9.62		
Psychiatry department	137	77.75 ± 11.53		
Emergency or Intensive care unit	19	72.68 ± 11.13		
Other departments	72	78.19 ± 10.58		
**Sleep Quality**			8.375^b^	0.000*
Poor	52	74.40 ± 8.35		
Common	167	76.04 ± 11.50		
Good	116	80.96 ± 9.74		
Extremely good	15	83.40 ± 9.73		
**Exercise frequency**			2.664^b^	0.048*
Never	77.39	77.39 ± 11.68		
Rarely	76.74	76.74 ± 10.57		
Often	80.05	80.05 ± 10.26		
Frequently	83.71	83.71 ± 13.65		
**Nurse-patient relationship**			-5.457^a^	0.000*
Common	75	71.96 ± 10.47		
Harmonious	275	79.32 ± 10.32		
**Whether felt tired when dealing with nurse-patient relationship**			13.309^b^	0.000*
Never	51	84.45 ± 10.67		
Rarely	116	79.28 ± 10.01		
Often	170	75.15 ± 10.47		
Frequently	13	71.69 ± 6.93		
**Whether felt overworked**			11.543^b^	0.000*
Never	21	85.19 ± 10.49		
Rarely	74	82.30 ± 10.40		
Often	199	75.63 ± 10.55		
Frequently	56	76.45 ± 9.23		

Note: ^a^ indicates the independent-sample t test; ^b^ indicates one-way analysis of variance; * indicates statistically significant difference (P < 0.05)

**Table 2:** Correlation between nurses’ parent-child relationships and other factors

	Family cohesion	Family adaptability	Age	Work seniority	The age of younger children	The age of older children
**Parent-child relationship** r(p)^a^	0.416 (< 0.001)	0.447 (< 0.001)	-0.116 ( 0.030)	-0.073 (0.174)	-0.090 (0.093)	-0.171 (0.001)

. Note: ^a^*r* Pearson Correlation Coefficient, *P P*-Value (Correlation is significant at the 0.05 level)

### Multivariate analysis of parent-child relationship in nurses

Statistically significant variables (P < 0.05), shown by t-tests, ANOVA, and correlation coefficients, were selected as the independent variables, and the parent-child relationship score was selected as the dependent variable to construct a multiple linear regression equation. The assignment of the independent variables is shown in Table 3. As shown in Table 4, the results showed that character (β = 0.143, P = 0.002), feelings of tiredness after dealing with patients (β=-0.150, P = 0.002), nurse-patient relationships (β = 0.137, P = 0.004), the age of older children (β=-0.153, P = 0.001), number of children (β=-0.093, P = 0.041), sleep quality (β = 0.116, P = 0.014), and family adaptability (β = 0.308, P = 0.000) were significant predictors of parent-child relationships, accounting for 31.3% of the total variation of the regression equation.

**Table 3:** Table of independent variable assignment

Independent variable	Assignment *(Dummy coded)*
Character	Uncertain (*Z1 = 0, Z2 = 0*); Introverted (*Z1 = 1, Z2 = 0*); Extroverted (*Z1 = 0, Z2 = 1*)
Relationship with spouse’s parents	Inharmonious = 1; Common = 2; Harmonious = 3
Number of children	Others (*Z1 = 0, Z2 = 0*); 1 child (*Z1 = 1, Z2 = 0*); 2 children (*Z1 = 0, Z2 = 1*)
Sleep Quality	Poor = 1; Common = 2; Good = 3; Extremely good = 4
Exercise frequency	Never = 1; Rarely = 2; Often = 3; Frequently = 4
Nurse-patient relationship	Common = 2; Harmonious = 3
Feelings of tiredness after dealing with patients	Never = 1; Rarely = 2; Often = 3; Frequently = 4
Whether felt overworked	Never = 1; Rarely = 2; Often = 3; Frequently = 4
Family cohesion	Bring in the original scores
Family adaptability	Bring in the original scores
Age	Bring in the original values
The age of older children	Bring in the original values

**Table 4:** Multivariate linear regression analysis of the factors influencing parent-child relationships

Variables	*B*	*SE*	*β*	*t*	*P*	*95% CI*
Family adaptability	0.473	0.074	0.308	6.389	0.000	0.328 ~ 0.619
Feelings of tiredness after dealing with patients	-2.069	0.673	-0.150	-3.074	0.002	-3.393~-0.745
Nurse-patient relationship	3.603	1.252	0.137	2.877	0.004	1.139 ~ 6.066
The age of older children	-0.357	0.106	-0.153	-3.367	0.001	-0.566~-0.149
Extroverted character (Reference: Uncertain)	3.087	0.990	0.143	3.118	0.002	1.139 ~ 5.034
Sleep quality	1.642	0.665	0.116	2.467	0.014	0.333 ~ 2.951
2 children (Reference: Others)	-2.267	1.105	-0.093	-2.051	0.041	-4.440~-0.093

Note: R^2^ = 0.326, adjusted R^2^ = 0.313, F = 23.665, P<0.001

## Discussion

To the best of our knowledge, this is the first large-sample study to evaluate the factors associated with parent-child relationships among Chinese nurses. Character, feelings of tiredness after dealing with patients, nurse-patient relationships, the age of older children, number of children, sleep quality and family adaptability were found to be significant predictors of the parent-child relationship in nurses.

In this study, the mean score for the parent-child relationship among nurses was 77.74 (SD = 10.77). In terms of the classification criteria of the scale score, this result suggests that although parent-child relationships in nurses were currently good, there was, nevertheless, need for improvement. Consistent with this finding, several other studies have also shown that parent-child relationships are generally good, with scores ranging from 75.21 to 77.93 [19, 40, 41]. Previous research has found that increased work intensity and workplace pressure lead to more distant parent-child relationships [3, 16]. Nurses experienced worse parent-child relationships due to the strain of their busy and long work schedules. One reason for the better scores observed here could be the protective effects of stability, regularity, and high salaries in the nursing profession [42]. Another reason may be that most of the children involved in this study were of preschool age, and thus may not have been fully exposed to the negative effects of nursing work. However, the findings of a recent study support our results, suggesting that increased work-family conflict does not affect the parenting styles of most parents [43]. In short, the impact of work on the family, especially the parent-child relationship, is not as simple as we thought, and the effective mechanism requires further investigation. Therefore, health policy-makers should actively support nurses with children, for example, by the provision of childcare assistance, flexible work schedules, and the development of strategies to strengthen the work and family interface of nurses, thus achieving effective human resource management. Meanwhile, organizing parent-child activities for the families of employees and promoting mutual assistance and support among employees would also be beneficial.

Through analysis, family adaptability was found to be the most important predictor of parent-child relationships, which is supported by the results obtained in previous studies [44, 45]. Stronger family adaptability would reduce authoritative behavior, fostering an open and harmonious family atmosphere that facilitates the making of joint decisions and participation in family affairs [45] In contrast, both parents that are too tolerant or have a strong desire for control can lead to children experiencing lower levels of family satisfaction and even psychological problems, indirectly affecting the maintenance of parent-child relationships [46, 47]. To promote and maintain balance within the family system, Olson & Gorall (2003) emphasized the importance of communication in the promotion of family adaptability, allowing family members to negotiate stability and change in a functional way [48]. As a result, after busy workdays, nurses should be encouraged to participate more in joint family decision-making and establish good relationships with their children, thus promoting good parenting and avoiding neglect of the child’s needs or the use of frequent punishment resulting from work-induced emotional exhaustion, physical, and mental fatigue, and other reasons.

The nurse-patient relationship of the participants was also identified as a factor that influenced the parent-child relationship; the present study is the first to report such findings. The relationship with patients appears to extend to the relationships with family members [49]. Maintaining a positive nurse-patient relationship allows nurses to develop empathy and manage emotions [50]. Likewise, positive interactions with patients can also help improve communication skills and better gain the trust of others [51]. These valuable experiences may gradually infiltrate and influence the parent-child relationship, resulting in a positive impact. Nevertheless, a poor nurse-patient relationship can exacerbate emotional exhaustion and even a lack of patience and confidence in dealing with family issues [52]. These may be the main reasons why nurse-patient relationships have a positive impact on parent-child relationships. Furthermore, the findings showed that nurses who experienced greater levels of tiredness resulting from nurse-patient relationships had poorer parent-child relationships. When dealing with nurse-patient relationships, nurses are often required to provide empathy and bear negative emotions [53]. In the absence of effective communication strategies and coping abilities, nurses are prone to emotional exhaustion and reduced levels of self-confidence [54]. This also applies to the maintenance of parent-child relationships. Obviously, this situation affects not only the way nurses interact with their children to a certain extent but also allows the accumulation of negative emotions that have nowhere to be released [48]. This may lead to emotional generalization in parenting, which would indirectly affect the parent-child relationship. Thus, health policy-makers should strive to create a supportive environment that allows nurses to express negative emotions and provide useful strategic guidance to nurses with tense nurse-patient relationships promptly, such as providing training in strategies to promote empathy and emotional management [50, 53]. In addition, nursing managers should also remind employees to avoid the transfer of negative work experiences to children.

The new insights derived from this study were that parent-child relationships of nurses deteriorated both with increased numbers of children and as the children grew older. This finding may be explained by the fact that as the number of children increases, parents may invest more time and energy in managing their children, which may lead to a shift from meticulous nurturing to lax management. Evidence has suggested that parents’ perceptions of child-rearing, children’s development,, and discipline vary greatly in relation to the number of children [55]. However, there is no consensus on the underlying mechanism by which the child’s age affects the parent-child relationship. One possible explanation is that while parents are satisfied with the relationships with their children and have more control when they are younger [56], as the children grow older, their dependence on their parents weakens, and parent-child conflicts become more frequent [57]. Thus, for nurses with children, especially those with multiple children, nursing managers should develop more flexible work schedules allowing them to cope with the care and management of their children. Furthermore, in order to better balance work-family conflict and take into account the healthy development of children, nursing managers should also consider providing training courses on developmental psychology for employees with multiple children or in cases of obvious parent-child alienation.

Interestingly, the study also found for the first time that extroverted nurses have better parent-child relationships. A previous study reported that extroverted mothers have a positive emotional core, high levels of activity, and interactions with other individuals that are regarded as pleasant, enjoyable, and satisfying, which supported this finding [58]. Parents with extrovert personalities contribute to positive communication and interaction between themselves and their children, improving the subjective well-being of the family [59]. Notably, Metsapelto & Pulkkinen (2005) stated that the children of such parents are more extroverted and self-assured and are more likely to feel the care and concern of extroverted parents [58]. In contrast, the children of introverted parents were more prone to frustration and conflict. As a result, while introverted nurses should be given more attention, transformation of their character should also be encouraged and guided.

Finally, the findings indicated that better sleep quality had a positive impact on the parent-child relationships of nurses. This may be because nurses who sleep poorly experience higher intensity workloads or more frequent night shifts, thereby reducing the time and effort invested in managing their children [9, 35] Fujimoto et al. (2008) found that three-shift duties are more likely to increase work-family conflict. Similarly, conflicts may escalate when there is a lack of support for early childhood care responsibilities in the workplace [9]. Thus, reasonable work intensity, longer shift work intervals, shift scheduling, and providing a healthy sleep plan in the workplace may help improve the sleep quality of nurses [60]. Another study suggested the use of exercise as a positive coping mechanism that may reduce stress levels and, therefore, have a positive impact on sleep quality [61]. To this end, nursing managers need to optimize the night-shift scheduling system to support nurses with children, while encouraging employees to engage in appropriate physical exercise.

### Limitations

This study had several limitations that were identified. First, the present study may be limited by the use of non-probabilistic sampling, which may affect the representativeness of the sample. Second, this study is a cross-sectional design and cannot determine causality as would a longitudinal study. Third, the children of nurses included in this study have a wide age range, which may affect the generalizability of the results; it is challenging to obtain data on nurses with children in a particular age group. Finally, all our subjects were from a large public hospital in Sichuan, China, and thus does not fully represent Chinese nurses.

## Conclusion

The findings of this study indicated that although the parent-child relationship among nurses is generally good at present, there is, nevertheless, need for improvement. The parent-child relationship in nurses was found to be related to several demographic characteristics (i.e., character, number of children, the age of older children). Thus, nursing managers should pay more attention to the parent-child relationships of nurses who are introverted and have more or older children. Furthermore, work-related factors, including nurse-patient relationships, feeling tired after dealing with nurse-patient relationships, and poor sleep quality, were also found to have effect on the parent-child relationships of nurses. Thus, health policy-makers should strive to create a family-oriented working environment, as well as provide timely preventive guidance and education for high-risk nurses with poor parent-child relationships identified in early screening. Another important finding of this study was that family adaptability was the most important predictor of parent-child relationships. After busy workdays, nurses should also be encouraged to participate more in family decision-making and establish good relationships with their children to avoid the negative effects work-related emotional exhaustion and physical and mental fatigue on the children. The development of good parent-child relationships may help maintain their and their children’s mental health while enhancing their enthusiasm for work and professional identity.

## Data Availability

I declare that all data and materials are available from the corresponding author upon reasonable request.
